# Resuscitative efficacy of hemoglobin vesicles for severe postpartum hemorrhage in pregnant rabbits

**DOI:** 10.1038/s41598-021-01835-w

**Published:** 2021-11-16

**Authors:** Hiroki Ishibashi, Kohsuke Hagisawa, Manabu Kinoshita, Yukako Yuki, Morikazu Miyamoto, Tomoko Kure, Hiromi Sakai, Daizoh Saitoh, Katsuo Terui, Masashi Takano

**Affiliations:** 1 Department of Obstetrics and Gynecology, National Defense Medical College, Tokorozawa, Saitama Japan; 2 Department of Physiology, National Defense Medical College, Tokorozawa, Saitama Japan; 3 Department of Immunology and Microbiology, National Defense Medical College, 3-2, Namiki, Tokorozawa, Saitama 359-8513 Japan; 4 Division of Anesthesiology, Saitama Medical University, Kawagoe, Saitama Japan; 5 Department of Chemistry, School of Medicine, Nara Medical University, Kashihara, Nara Japan; 6 Division of Traumatology, National Defense Medical College Research Institute, Tokorozawa, Saitama Japan

**Keywords:** Drug development, Experimental models of disease, Preclinical research

## Abstract

We aimed to investigate the resuscitative efficacy of hemoglobin vesicles (HbVs) as a red blood cell (RBC) substitute for the initial treatment of severe postpartum hemorrhage (PPH). Twenty-five pregnant rabbits underwent cesarean section; uncontrolled hemorrhage was induced by transecting the right uterine artery to establish a severe PPH model. During the first 30 min, all rabbits were administered 6% hydroxyethyl starch (HES) of an equivalent volume to the hemorrhage every 5 min. Thereafter, they received any of the following three isovolemic fluids for resuscitation every 5 min: RBCs with platelet-poor plasma (RBC/PPP) (*n* = 8), 6% HES (*n* = 7), or HbVs with 25% human serum albumin (*n* = 10). After surgical hemostasis at 60 min, survival was monitored until 12 h. No rabbits receiving only HES infusion survived beyond 6 h, whereas all rabbits receiving RBC/PPP transfusion survived. The rabbits receiving HbV infusion showed significantly higher mean arterial pressure and hemoglobin levels than the HES-receiving rabbits, and 8 of 10 rabbits survived for 6 h. The HbV group showed significantly higher survival than the HES group but worse survival than the RBC/PPP group. In conclusion, HbV infusion for severe PPH effectively prevents lethal hemorrhagic shock in a pregnant rabbit model.

## Introduction

Severe postpartum hemorrhage (PPH) remains the leading cause of maternal morbidity and mortality worldwide^1,2^. At least 26% of PPH-related deaths result from insufficient and delayed blood transfusion^3^, and thus timely blood transfusions are essential. However, access to blood markedly differs between low- and high-income countries. Transfusion for severe PPH commonly involves red blood cells (RBCs) and other blood components. Every obstetrical facility should have an emergency strategy for severe PPH, including protocols for accessing packed RBCs^4^. However, blood transfusion services are highly resource intensive and require voluntary donations; donor screening, including blood-type antigen and cross-matching test; and a temperature-controlled system. Furthermore, the spread of epidemics, such as the COVID-19 infection, limits the available blood supply worldwide^5,6^. Thus, preparing adequate resources at small facilities is challenging.

To support the system, hemoglobin (Hb)-based oxygen carriers (HBOCs) were developed as blood substitutes for RBCs^7^. Cell-free typed HBOCs exert some side effects of bared Hbs, including nitric oxide (NO_2_^−^)-related vasoconstriction, hypertension, and higher infarction rates^8^. These results emphasize the significance of mimicking the cellular structure of RBCs. Therefore, many studies have attempted Hb encapsulation using liposomes to improve biocompatibility, storage stability, and oxygen-carrying capacity. Accordingly, hemoglobin vesicles (HbVs) were developed^9^. The liposomes in the structures of HbVs prevent the release of free Hb and avoid NO_2_^–^related vasoconstrictive complications^10–14^. Blood substitutes including HbVs could be useful for hemorrhagic shock^15–18^. Furthermore, HbVs could be stored for at least 1 year; do not need donor screening, including blood-type antigen or cross-matching test; and have no risk of blood contamination^17,18^.

Severe PPH is a type of hemorrhagic shock, and its treatment is basically similar to that of hemorrhagic shock^19^. However, no study has assessed the efficacy of HbVs for severe PPH. Moreover, animal models have been limited. Yu et al.^20^ created a hemorrhagic shock model in pregnant rabbits; however, it was not a PPH model. This study aimed to investigate the resuscitative efficacy of HbVs for PPH. Toward this goal, we established a severe PPH model using pregnant rabbits based on top reflect clinical scenarios. Then we hypothesized in this study that HbVs could have a resuscitative effect on severe PPH as an alternative to blood transfusion.

## Methods

### Animal management

Female New Zealand white rabbits (weight: 3.5–3.9 kg, gestational age: 28 days [normal gestation period, 29–35 days]); Japan SLC, Hamamatsu, Japan) were used. They were given free access to standard feed and water during a 7-day adaptation period before the experiment. Male New Zealand white rabbits (weight: 2.6–2.8 kg; Japan SLC, Hamamatsu, Japan) were used as blood donors. The experimental protocol was approved by the Institutional Review Board for the Care of Animal Subjects of the National Defense Medical College (ethical approval number: #17032). All applicable international, national, and/or institutional guidelines for the care and use of animals were followed with strict adherence to ARRIVE guidelines^21,22^ (Supplementary Note online).

### Preparation of hemoglobin vesicles

HbVs were prepared as previously described^15,16^. Briefly, Hb was purified from outdated donor human blood provided by the Japanese Red Cross Society. Encapsulated carbonylhemoglobin (HbCO, 38 g/dL) contained 5.9 mM pyridoxal 5″-phosphate (Sigma Chemical, Saint Louis, USA) as an allosteric effector for regulating oxygen affinity. The lipid bilayer was a mixture of 1,2-dipalmitoyl-sn-glycero-3-phosphatidylcholine, cholesterol, and 1,5-bis-O-hexadecyl-N-succinyl-l-glutamate at a molar ratio of 5:4:0.9 and 1,2-distearoyl-sn-glycero-3-phosphatidyl-ethanolamine-N- polyethylene glycol (PEG) (0.3 mol%). HbV particles were suspended in isotonic saline; nitrogen gas was bubbled through the solution in a vial to remove the oxygen for ensuring product stability. Then, HbVs were stored in deoxygenated glass vials at 4 °C for 10–12 months. The HbV properties used in this experiment are shown in Table 1. The HbV solution showed no colloid osmotic pressure^23^, similar to RBCs when suspended in isotonic saline. Before the experiments, the HbV solution was mixed with 25% human serum albumin, which was concentrated by 5–6 times more than plasma (Benesis, Osaka, Japan) (vol/vol = 4:1), to adjust the albumin concentration of the vesicle-suspension medium to 5 g/dL and the colloid osmotic pressure to approximately 20 mmHg same as plasma.

**Table 1:** Properties of hemoglobin vesicles.

Solution	pH	Hemoglobin (g/dL)	Lipids (g/dL)	P50* (mmHg)	Particle diameter (nm)	Methemoglobin (%)
HbVs	6–8	10	8–10	15–20	250–280	8–10

Data are expressed as the value or range.

*p50, arterial blood oxygen tension at which hemoglobin is half saturated with oxygen.

*HbVs* hemoglobin vesicles.

### Preparation of allogenic RBCs

Donor rabbits (*n* = 16) were anesthetized using an intramuscular injection of 25 mg/kg ketamine and 10 mg/kg xylazine. A 20-gauge catheter (polyethylene indwelling needle; Terumo, Tokyo, Japan) was aseptically introduced into the left femoral artery for blood sampling. Then, 50 mL/kg of donor blood was withdrawn from this artery. After removing platelet-poor plasma (PPP) from the blood by centrifugation (100×*g* for 15 min), the remaining RBCs were washed with acid citrate dextrose solution^16^. Subsequently, the same volume of mannitol adenine phosphate solution (1.457% [w/v%] d-mannitol, 0.014% adenine and 0.094% sodium dihydrogen phosphate [Terumo, Tokyo, Japan]) was added to prepare allogenic RBCs that were then stored at 4 °C. Before usage, we performed a cross-matching test between donor RBCs and recipient plasma and a test between donor plasma and recipient RBCs, same as clinical examination. PPP samples showed the following coagulation activity: fibrinogen, 121 mg/dL; antithrombin (AT) III activity, 81%; coagulation factor VIII, 388 ng/mL; prothrombin time (PT), 13.1 s; and activated partial thromboplastin time (APTT), 31.2 s, on average. Rabbit coagulation factor VIII in the rabbit PPP was measured using the Rabbit coagulation factor VIII ELISA kit (abx363389, Abbexa Ltd, Cambridge, UK). We showed the data of these coagulation parameters of rabbit PPP in Table 2.

**Table 2:** Values in coagulation parameters of rabbit PPP.

PT (s)	APTT (s)	Fibrinogen (mg/dL)	AT III (%)	Factor VIII (ng/mL)
12.2 ± 0.9	31.2 ± 1.7	133 ± 21	85 ± 11	387.8 ± 121.5

Data are expressed as the mean ± standard deviation.

### Surgical procedures

Pregnant rabbits were anesthetized using an intramuscular injection of 25 mg/kg ketamine and 10 mg/kg xylazine, followed by intravenous injections of 15 mg/kg pentobarbital. Anaesthesia was maintained with additional doses as necessary. A local anaesthetic (3–5 mL of 1% lidocaine) was injected subcutaneously into the left inguinal area and mid-lower abdomen. The adequacy of general anaesthesia was monitored according to the loss of the ear pinch reflex. Rabbits were maintained in the supine position and were spontaneously breathing (room air) on the warming plate to maintain the body temperature at a range of 36–37 °C. A 20-gauge catheter (polyethylene indwelling needle; Terumo, Tokyo, Japan) was introduced into the left femoral artery for measuring blood pressure and heart rate and blood sampling. A same sized catheter was similarly inserted into the left femoral vein for fluid injections. After instrumentation, the rabbits were stabilized for 10 min to record baseline data.

### Severe postpartum haemorrhage model

**Figure 1:**
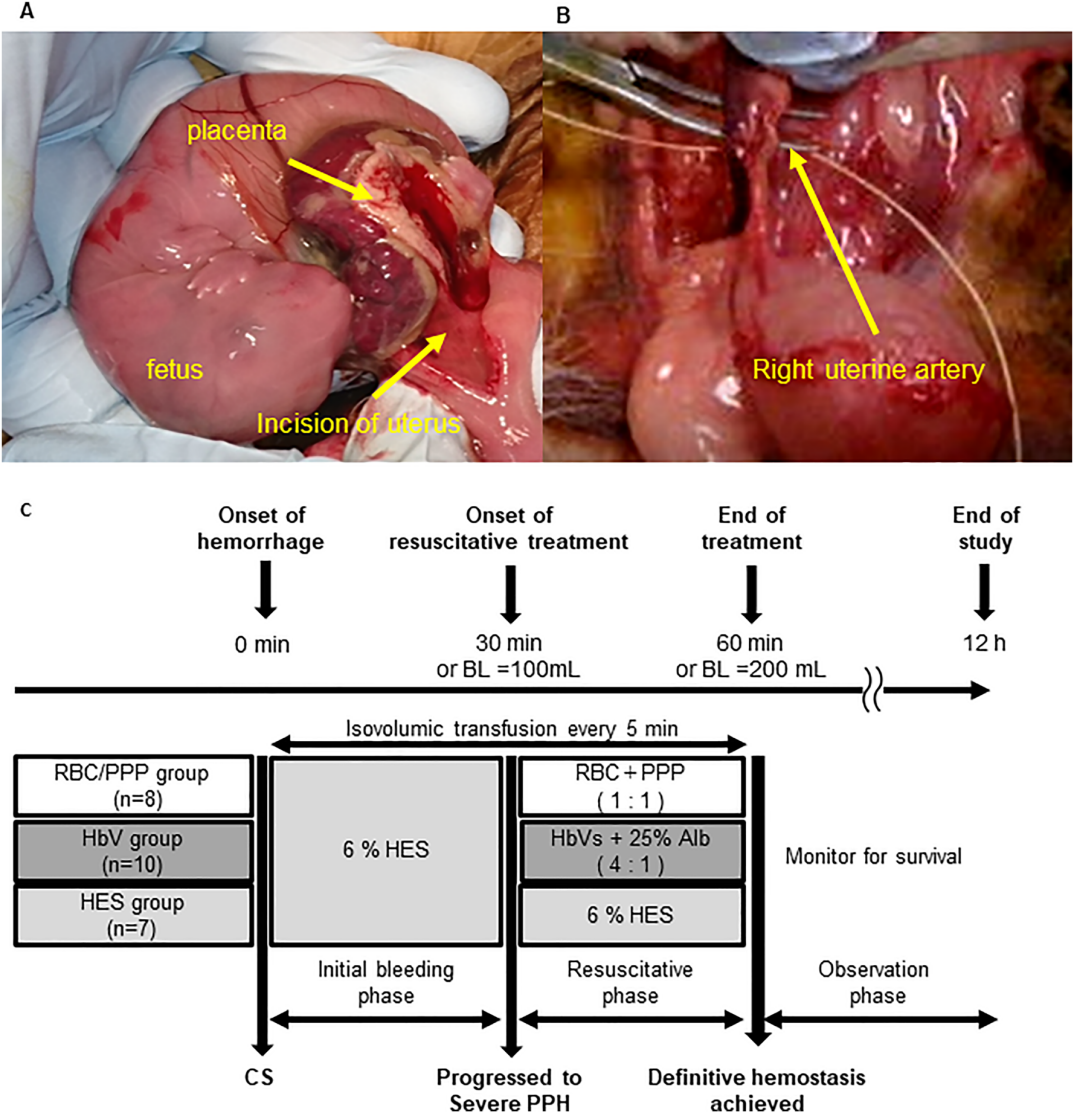
Surgical procedures and experimental protocol. (**A**) Rabbits underwent cesarean section to deliver fetuses from the right side of the bicornate uterus. (**B**) After closing the incision in the uterus, uncontrolled hemorrhage is induced by transecting the right uterine artery. (**C**) Pregnant rabbits undergo CS, and uncontrolled hemorrhage is progressed to severe PPH. First, all rabbits are administered 6% HES for initial treatment. After progressed to severe PPH, the rabbits receive isovolemic fluid resuscitation with an equivalent volume of hemorrhage every 5 min. Resuscitative administration regimens include the following: RBC/PPP (vol/vol = 1:1, *n* = 8), 6% HES (*n* = 7), or HbVs with 25% human serum albumin (vol/vol = 4:1, *n* = 10). After 60 min from the start of hemorrhage or when the hemorrhage volume reaches 200 mL, surgical hemostasis is performed. Survival is monitored thereafter for at least 12 h. Data shown are the mean ± standard deviation. *Alb* albumin, *BL* blood loss, *CS* caesarean section, *HbV* haemoglobin vesicles, *HES* hydroxyethyl starch, *PPH* postpartum haemorrhage, *RBC/PPP* red blood cells with platelet-poor plasma.

We designed the severe PPH model and the actual clinical treatment after delivery. After lower midline laparotomy, we performed a caesarean section and delivered foetuses from the right side of the rabbit bicornuate uterus (Fig. 1A). Thereafter, uncontrolled haemorrhage was induced by transecting the right uterine artery (Fig. 1B). The bleeding was wiped off using a high-absorbent gauze (BEMCOT M-1, Asahikasei, Tokyo, Japan), and the absorbed blood volume was assessed based on the weight of gauze every 5 min. First, to compensate for the initial bleeding, an isovolemic infusion of 6% hydroxyethyl starch (HES) (Voluven 6%; Fresenius Kabi Deutschland, Bad Homburg, Germany) was repeated every 5 min through the femoral vein until 30 min or the bleeding volume reached 100 mL. The reason for setting the bleeding volume at 100 mL is as follows.

The need for blood transfusion was determined when the shock index (heart rate/systolic blood pressure) exceeded 1.5 based on a previous study, with a shock index of 1.5 corresponding to a blood loss volume of approximately 2.5 L^24^. In pregnant women weighing 60 kg, the circulating blood volume is approximately 4.6 L; thus, the blood loss rate in severe PPH is estimated at approximately 54% of circulating blood. According to the guidelines of the European Federation of Pharmaceutical Industries Associations and the European Centre for the Validation of Alternative Methods^25^, rabbit circulating blood is approximately 56 mL/kg. Considering that pregnant rabbits weighed approximately 3.5 kg, the bleeding volume corresponding to severe PPH was calculated at 106 mL (3.5 kg × 56 mL/kg × 0.54). Thus, we set the bleeding volume of severe PPH at 100 mL.

### Fluid resuscitation with haemoglobin vesicles, RBCs, or 6% HES

Following the initial infusion of 6% HES, rabbits were randomized into three groups using the random function in Microsoft Excel (Supplementary Note online After progression to severe PPH, the rabbits in each group received an isovolemic fluid (allogenic RBC with PPP, 6% HES, or HbVs with 25% human serum albumin) for resuscitation, which was equivalent to the blood loss every 5 min until 60 min or when the bleeding volume reached over 200 mL (Fig. 1C), similar to the circulation volume in rabbits^23^. In pregnancy, such condition is lethal hemorrhagic shock class IV^26^. The fluid regimens consisted of allogenic RBC with PPP (vol/vol = 1:1, Hb concentration of 11.9 ± 1.6 g/dL) (RBC/PPP group, *n* = 8) as a positive control, 6% HES infusion (HES group, *n* = 7) as a negative control, and HbVs with 25% human serum albumin (vol/vol = 4:1, Hb concentration of 8.0 g/dL) (HbV group, *n* = 10) (Fig. 1C). The selection of resuscitation fluid is controversial, and saline is the most commonly used fluid. Colloid solutions such as hydroxyethyl starch are possibly harmful in some patients. In contrast, these colloid solutions are used for many patients and many resuscitation episodes, including hemorrhagic shock, in several countries^27^. We selected HES infusion as a negative control for HbV infusion. After fluid resuscitation, the rabbits underwent a surgical hemostatic procedure via the ligation of a bleeding right uterine artery, followed by cesarean section to deliver the fetuses from the left side of the bicornate uterus. The laparotomy incisions were closed, and survival was monitored for at least 12 h. Postoperative analgesia was induced with intramuscular injections of buprenorphine (0.02 mg/kg) after 12 h for euthanasia.

### Measurements of blood cell counts, coagulation factors, and blood gases

Blood samples were obtained every 5 min for blood cell counting using an Erma PCE 170 hematology analyzer (Erma, Tokyo, Japan). Coagulation factors, except d-dimers, and blood gases were examined every 15 min. d-dimer levels were examined at 60 min. Hb concentration in the blood containing HbVs could not be accurately determined using the current analyzer because liposome capsules interfered with the spectrophotometric measurement of Hb absorbance. The actual Hb concentrations were estimated using a previous method^16^. To measure plasma AT III activity, PT, APTT, and D-dimers, blood samples were collected with a 3.2% sodium citrate solution and centrifuged at 50,000×*g* at 4 °C for 20 min to remove HbV particles. Measurements were conducted at Sanritsu Zelcova Laboratory (Tokyo, Japan). Plasma fibrinogen levels were measured using a rabbit fibrinogen enzyme-linked immunosorbent assay kit (LifeSpan BioSciences, Seattle, USA). Blood gas analyses, including plasma lactate and arterial oxygen content (CtO_2_) levels, were performed using an ABL 80 blood gas analyzer (Radiometer, Copenhagen, Denmark).

### Analyses of whole blood coagulation activity and plasma nitric oxide

Coagulation activity was measured every 15 min using a coagulation and platelet (PLT) function analyzer (Sonoclot) (Sienco Inc, Morrison, CO). The Sonoclot signal typically describes coagulation parameters, including activated clotting time (ACT) (which indicates the period up to the start of fibrinogen formation) and clot rate. Plasma NO_2_^-^ levels were measured every 15 min. Blood samples were collected with a heparinized syringe and centrifuged at 50,000×*g* at 4 °C for 20 min to remove HbV particles. The supernatant was stored at − 80 ℃ until analysis. Plasma NO_2_^-^ levels were measured using a high-performance liquid chromatography-Griess system in Eicom Laboratory (Eicom Inc Co, Kyoto, Japan).

### Plasma biochemical test

To evaluate haemolysis and acute kidney injury, plasma creatinine, iron, cell-free Hb, and cell-free heme levels were measured before the experiment and 60 min after bleeding. Plasma creatinine levels were measured using the Fuji Dry-Chem system (Fujifilm Medical, Saitama, Japan). Plasma iron levels were measured at Sanritsu Zelcova Laboratory (Tokyo, Japan). Plasma free Hb levels were measured using Plasma/Low Haemoglobin Hemocue (HemoCueAB, Angelholm, Sweden). Plasma cell-free heme levels were measured using the heme assay kit (BioChain Institute Inc, Newark, USA). All plasma samples were stored at − 80 ℃ until analysis.

### Statistical analysis

The sample size for each group complied with the ARRIVE guidelines^21,22^ (Supplementary Note online). In addition, statistical power analysis was performed using G*Power, version 3.1.9.4 software (https://www.psychologie.hhu.de/arbeitsgruppen/allgemeine-psychologie-und-arbeitspsychologie/gpower.html)^28^. Survival curves were generated using the Kaplan–Meier method and compared using the log-rank test. Statistical comparisons between two groups and among three groups were conducted using Student’s t-test and one-way analysis of variance, followed by the Bonferroni post hoc test, respectively. Data are presented as the mean ± standard deviation (SD). All statistical analyses were performed using JMP software Pro 14.0.0 (SAS Institute Inc., Tokyo, Japan, https://www.jmp.com/ja_jp/software/data-analysis-software.html). P < 0.05 was considered statistically significant.

## Results

### Hemodynamic changes and rabbit survival

**Figure 2:**
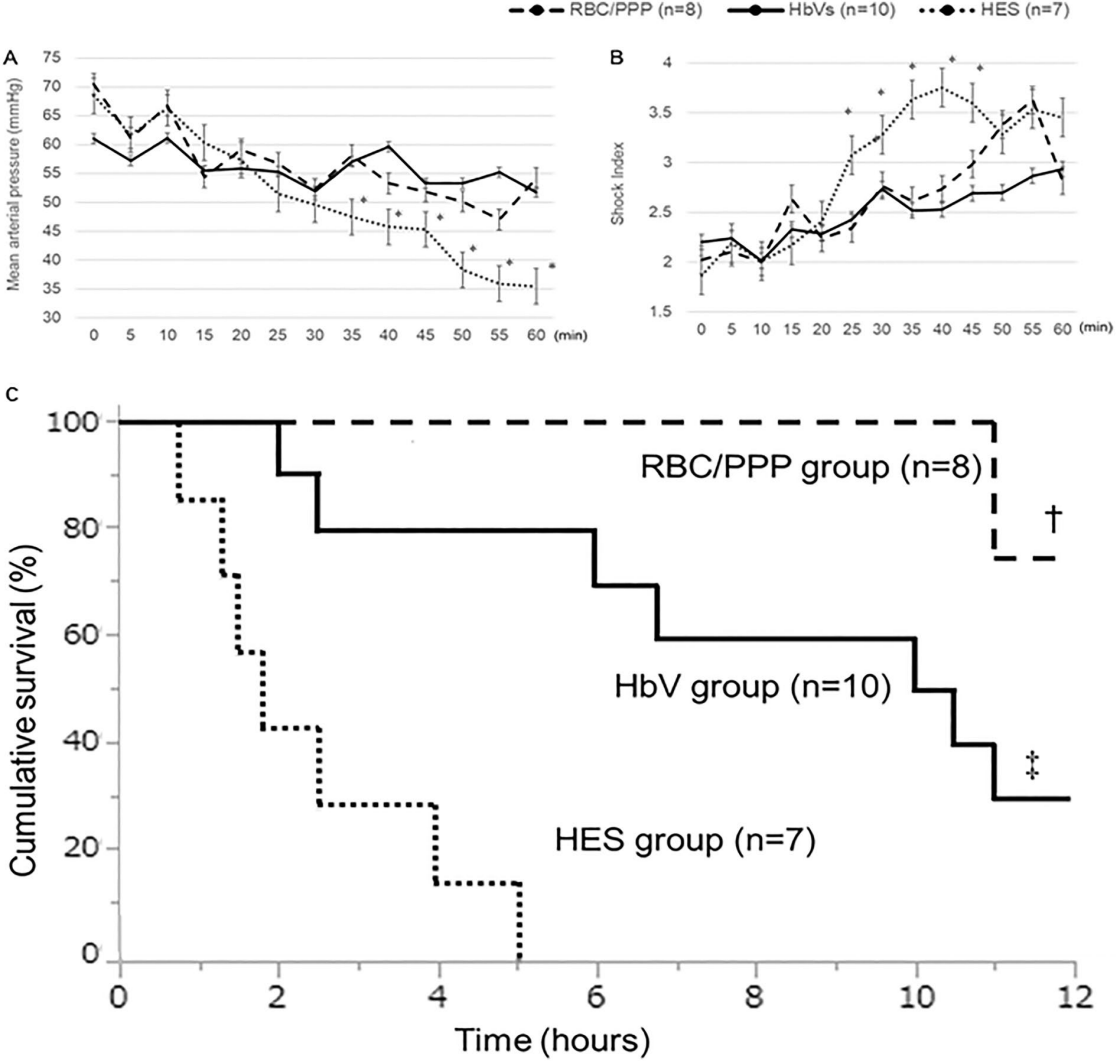
Hemodynamic changes and survival curves of all three groups. (**A**) The HES group shows progressively decreased MAP after 30 min, and the values are significantly lower than in the other groups. (**B**) In the HES group, the shock index from 25 to 45 min is significantly higher than in the other groups, whereas there is no significant difference at 60 min. (**C**) Survival of the HbV group is significantly better than that of the HES group (P < 0.01), whereas it is significantly worse than that of the RBC/PPP group (P = 0.01). *P < 0.05, value significantly different from the other groups. ^†^P < 0.05, the prognosis of the RBC/PPP group is significantly different from that of the other groups. ^‡^P < 0.05, the prognosis of the HbV group is significantly different from that of the HES group. Data shown are the mean ± standard deviation. *HbV* hemoglobin vesicles, *HES* hydroxyethyl starch, *MAP* mean arterial pressure, *RBC/PPP* red blood cells with platelet-poor plasma.

The rabbits developed postoperative severe anemia (Hb concentration < 6 g/dL) that progressed to severe PPH in the first 30 min. The time to severe PPH (bleeding volume > 100 mL) was 23 ± 7 min in the HES group, 24 ± 5 min in the HbV group, and 23 ± 4 min in the RBC/PPP group (Table 3), with no significant differences among the groups. There were also no significant differences in the total hemorrhage volume and total bleeding time (Table 3). Although the HES group showed progressively decreased mean arterial pressure (MAP) < 40 mmHg in the resuscitative phase (beyond 30 min), HbV and RBC/PPP groups maintained significantly higher MAP than the HES group in this period (P < 0.05) (Fig. 2A). Shock index was gradually increased in all groups; the shock index at 25–45 min was significantly higher in the HES group than in the other groups (P < 0.05), whereas no significant differences were observed among the three groups at 60 min (Fig. 2B). Within 6 h, all rabbits in the HES group died, whereas all rabbits in the RBC/PPP group survived, and 8 of the 10 rabbits in the HbV group survived. Regarding overall survival, the prognosis of the HbV group was significantly better than that of the HES group (P < 0.01), whereas it was significantly worse than that of RBC/PPP group (P = 0.01) (Fig. 2C).

**Table 3:** Variables associated with hemorrhage.

Variables	HES group *n* = 7	HbV group *n* = 10	RBC/PPP group *n* = 8	*p*
Time to severe PPH (min)	23 ± 7	24 ± 5	23 ± 4	n.s
Total hemorrhage (mL)	211 ± 34	203 ± 54	199 ± 41	n.s
Total bleeding time (min)	44 ± 14	49 ± 11	50 ± 13	n.s

Data are expressed as the mean ± standard deviation.

*HbV* hemoglobin vesicle, *HES* hydroxyethyl starch, *n.s* not significant, *PPH* postpartum hemorrhage, *RBC/PPP* red blood cells with platelet-poor plasma.

### Hematologic variables

**Figure 3:**
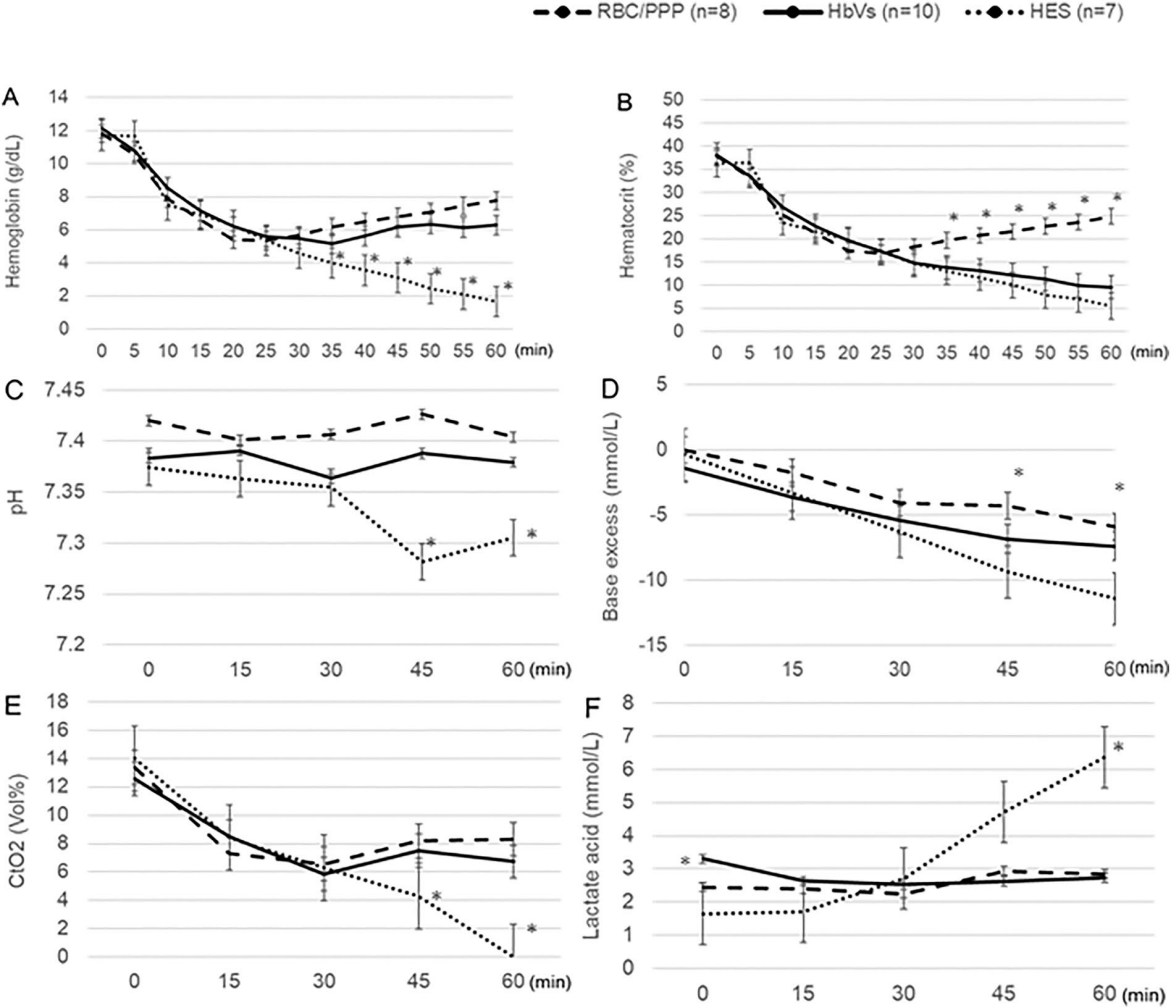
Changes in the levels of hematologic and blood gas variables. (**A**) At 60 min, the hemoglobin concentration is maintained over 6.0 g/dL in the RBC/PPP and HbV groups, whereas it decreases under 2.0 g/dL in the HES group. (**B**) The hematocrit is similarly decreased in both the HbV and HES groups, unlike that in the RBC/PPP group. (**C**) The pH levels in the HES group are significantly lower than those in the other groups after 30 min. (**D**) The base excess levels at 45 and 60 min are significantly higher in the RBC/PPP group than in the other groups. (**E**) The CtO_2_ level is decreased in all groups until 30 min; however, it is maintained above approximately 7.0 vol% beyond 30 min in the RBC/PPP and HbV groups. (**F**) At 60 min, the lactate acid levels are significantly higher in the HES group than in the other groups. *P < 0.05, value significantly different from the other groups. Data shown are the mean ± standard deviation. *CtO*_*2*_ concentration of total oxygen, *HbV* hemoglobin vesicles, *HES* hydroxyethyl starch, *RBC/PPP* red blood cells with platelet-poor plasma.

The administration of HbVs and RBC/PPP following the initial HES infusion gradually increased Hb concentration, and it was maintained over 6 g/dL in both groups, though the Hb concentration decreased under 2 g/dL at 60 min in the HES group (Fig. 3A). Unlike the Hb dynamics, the decrease in hematocrit (Hct) was similar between the HbV and HES groups, although the Hct level was higher in the RBC/PPP group (Fig. 3B). PLT counts could not be measured in the HbV group because the submicron HbV particle interfered with PLT counting with the current analyzer. Meanwhile, PLT counts gradually decreased in the RBC/PPP group but remained significantly higher than those in the HES group at 60 min (Supplementary Fig. S1online).

### Blood gas analyses

**Figure 4:**
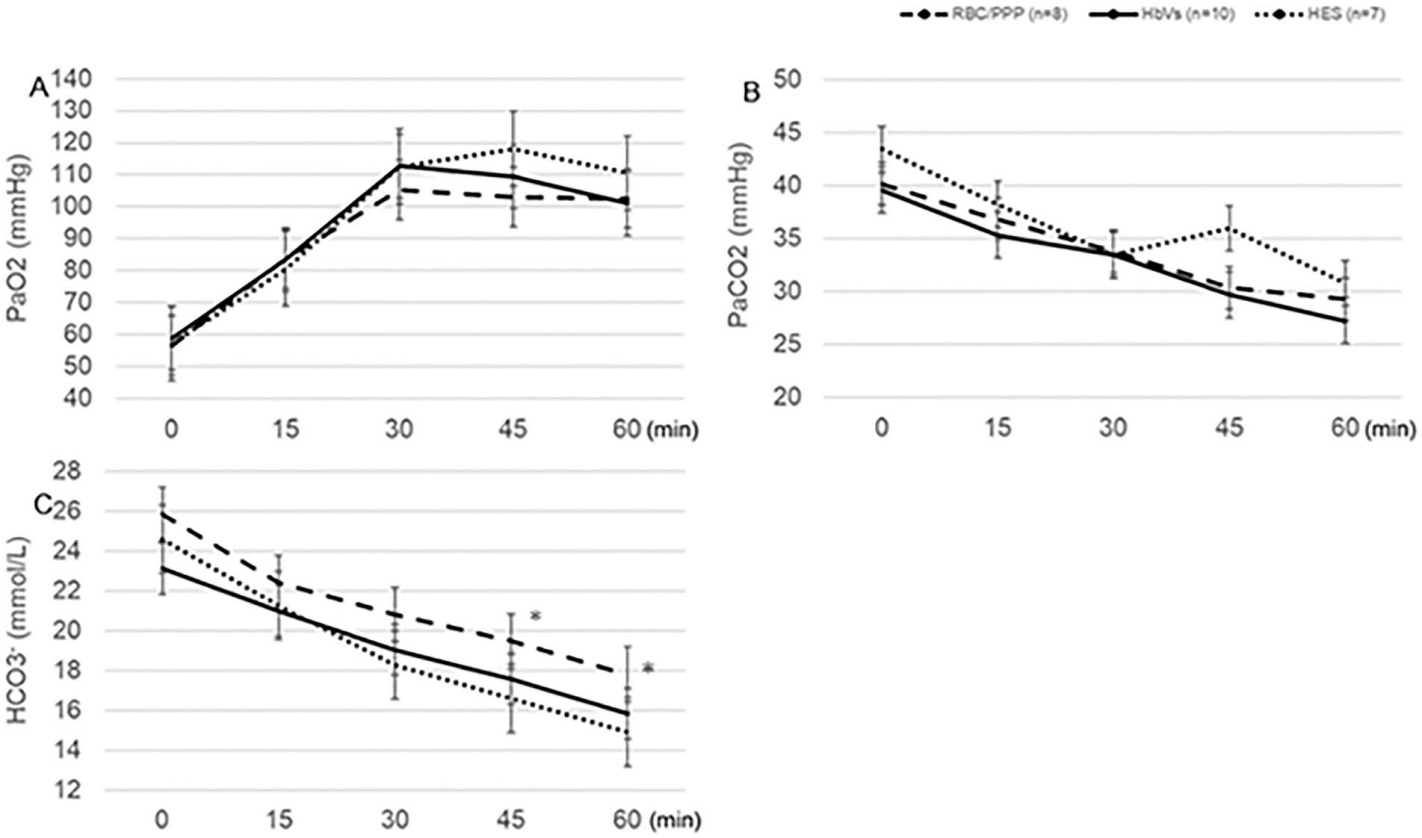
Changes in the levels of (**A**) PaO_2_, (**B**) PaCO_2_, and (**C**) HCO_3_. (**A**) There are no significant differences in the PaO_2_ levels among all the groups throughout the experiment. (**B**) There are also no significant differences in the PaCO_2_ levels among all groups throughout the experiment. (**C**) At 45 and 60 min, the HCO3^-^ level is significantly higher in the RBC/PPP group than in the other groups. *P < 0.05, value significantly different from the other groups. Data shown are the mean ± standard deviation. *HbV* hemoglobin vesicles, *HES* hydroxyethyl starch, *RBC/PPP* red blood cells with platelet-poor plasma.

The pH level was maintained above 7.35 in the HbV and RBC/PPP groups, whereas it was significantly decreased below 7.35 at 45 and 60 min in the HES group, which was significantly lower than in the other groups (P < 0.01) (Fig. 3C). The base excess and HCO_3_^-^ levels were significantly higher in the RBC/PPP group than in the other groups at 45 and 60 min (P < 0.05) (Figs. 3D and 4C). In contrast, no significant differences in the values of PaO_2_ or PaCO_2_ were observed among the groups throughout the experiment (Fig. 4A,B). During the initial 30 min, the concentration of total oxygen (CtO_2_) (i.e., the sum of oxygen bound to Hb) decreased to approximately 6.5 vol% in all the three groups. RBC transfusion or HbV infusion restored the CtO_2_ to 8.0 vol%. In contrast, the CtO_2_ decreased to unmeasurable values at 60 min in the HES group (Fig. 3E). The lactate levels at baseline in the HbV group was statistically higher than that in other groups, but this value was clinically within the normal range. Meanwhile, lactate levels were maintained at approximately 2.0- 3.0 mmol/L throughout the experiment in the HbV and RBC/PPP groups, whereas it was critically increased to 6 mmol/L at 60 min in the HES group, which was significantly higher than that in the other groups (P < 0.05) (Fig. 3F).

### Coagulation variables and activity

**Figure 5:**
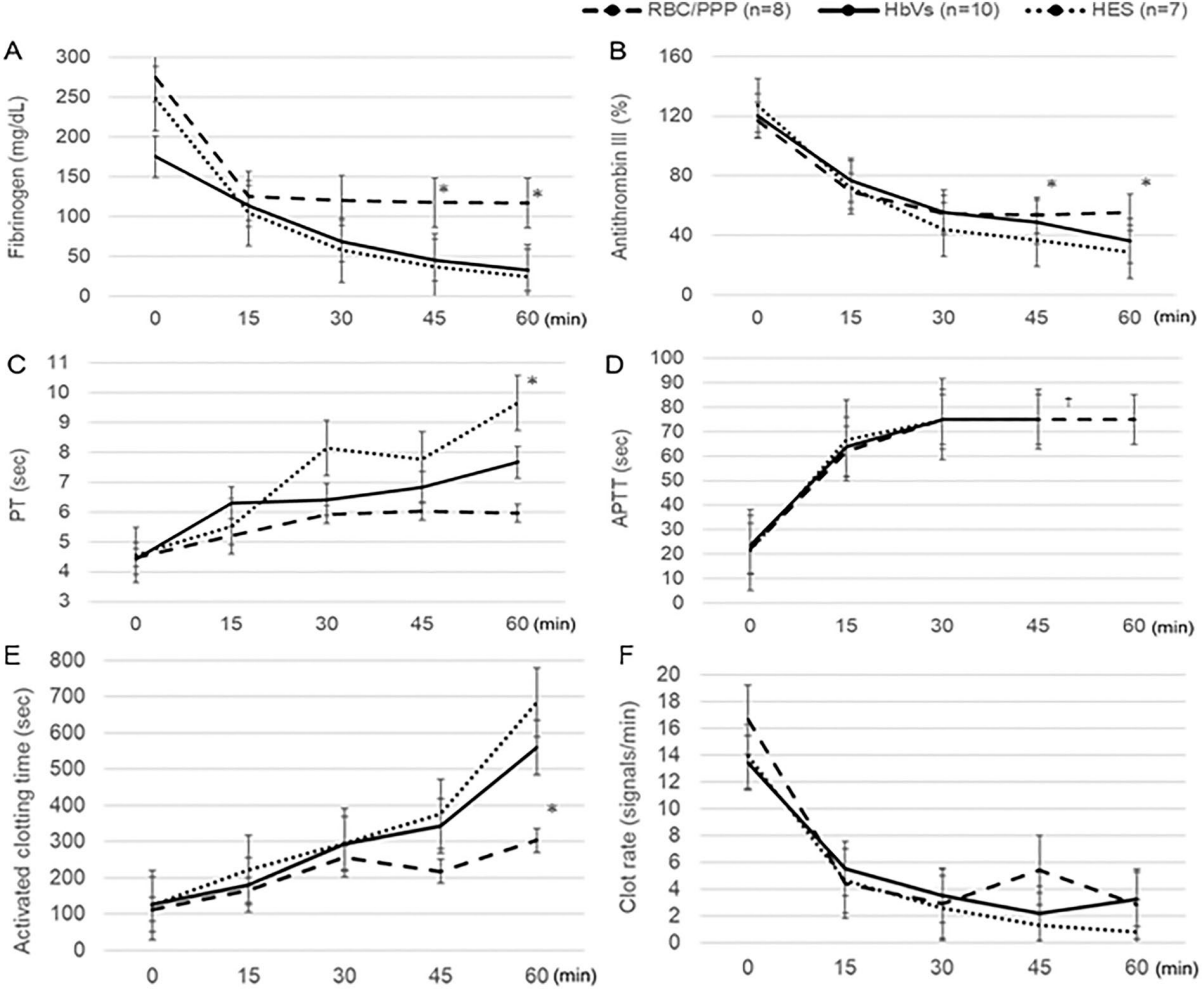
Changes in the levels of coagulation variables. (**A**) The plasma fibrinogen values in the RBC/PPP group are maintained over 110 mg/dL even at 60 min. (**B**) The AT III values are significantly lower in the HbV and HES groups than in the RBC/PPP group at 45 and 60 min. (**C**) The PT values are significantly longer in the HbV and HES groups than in the RBC/PPP group at 60 min. (**D**) The APTT values after 30 min is set as the reference value because the value over 75 s could not be measured in the current analysis. (**E**) ACT at 60 min is significantly longer in the HES group and the HbV group than in the RBC/PPP group. (**F**) There are no significant differences in the clot rate among the three groups throughout the experiment. *P < 0.05, value significantly different from the other groups. ^†^At 60 min, the APTT levels are unmeasurable in the HbV and HES groups. Data shown are the mean ± standard deviation. *ACT* activated clotting time, *APTT* activated partial thromboplastin time, *AT* *III* antithrombin III, *HbV* hemoglobin vesicles, *HES* hydroxyethyl starch, *PT* prothrombin time, *RBC/PPP* red blood cells with platelet-poor plasma.

The plasma fibrinogen levels in the RBC/PPP group gradually decreased but was maintained over 110 mg/dL even at 60 min because PPP contained a certain amount of coagulation factors, including fibrinogen (Fig. 5A). In contrast, the plasma fibrinogen levels continuously decreased and reached 20 mg/dL in both the HbV and HES groups (Fig. 5A). The values of AT III in both the HbV and HES groups were significantly lower than those in the RBC/PPP group at 45 (P < 0.05) and 60 min (P < 0.01) (Fig. 5B). Similarly, PT was significantly longer in the HbV and HES groups than in the RBC/PPP group at 60 min (P < 0.01) (Fig. 5C). APTT was prolonged beyond 75 s at 30–60 min in all three groups (Fig. 5D). In addition, plasma D-dimers levels were not elevated in any groups (< 0.2 μg/mL) at 60 min (not shown).

Regarding coagulation activity, ACT at 60 min was significantly longer in both the HbV and HES groups than in the RBC/PPP group (P < 0.05) (Fig. 5E). Regarding the clot rate, which indicated the slope of fibrin gel formation that was affected by both fibrinogen to fibrin conversion and the amount of fibrinogen, all three groups showed similar decreases in the clot rate with no significant differences (Fig. 5F).

### Changes in the plasma NO_2_^−^ level and biochemical status

**Figure 6:**
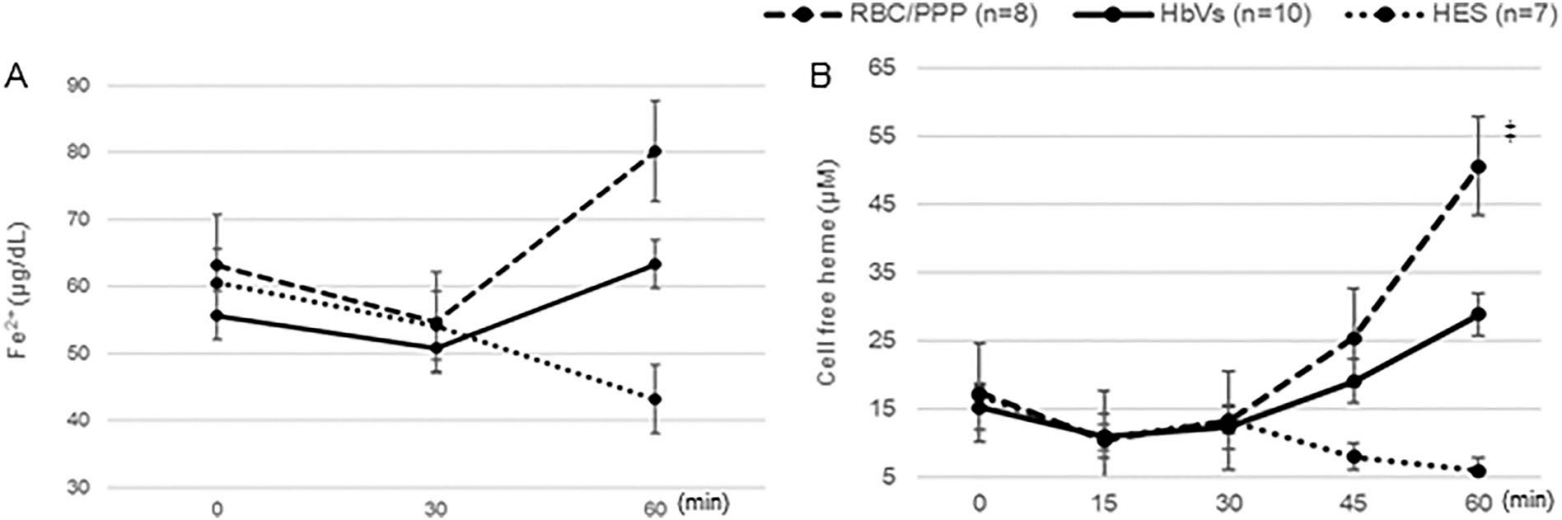
Changes in the levels of Fe^2+^ and cell-free heme. (**A**) The values of Fe^2+^ tends to increase in the HbV and RBC/PPP groups after 30 min. (**B**) The concentration of cell-free heme is increased after RBC transfusion or HbV infusion, with the increase being greater after RBC transfusion than after HbV infusion. ^‡^P < 0.05, The concentration of cell-free hemoglobin increased after RBC transfusion than after HbV infusion. Data shown are the mean ± standard deviation. *HbV* hemoglobin vesicles, *HES* hydroxyethyl starch, *RBC* red blood cell, *RBC/PPP* red blood cells with platelet-poor plasma.

The HbV group did not show a significant reduction in NO_2_^−^ levels (Table 4). HbV infusion did not induce hemolytic and acute renal dysfunction as indicated by the values of iron and creatinine being maintained within normal ranges. In addition, there was no significant increase in free Hb levels (Fig. 6A, Table 4). Meanwhile, although the cell-free heme levels were higher at 60 min after RBC transfusion or HbV infusion than at baseline, the increase in cell-free heme at 60 min was significantly lower in HbV infusion than in RBC transfusion (P < 0.05) (Fig. 6B).

**Table 4:** Plasma levels of nitric oxide, creatinine, and free Hb at 60 min by group.

Variables	Baseline	HbV group (n = 10)	RBC/PPP group (n = 8)	HES group (n = 7)
NO_2_^-^ (μM)	0.9 ± 0.4	0.7 ± 0.3	1.0 ± 0.3	0.7 ± 0.4
Creatinine (mg/dL)	0.9 ± 0.1	0.9 ± 0.2	0.9 ± 0.2	0.9 ± 0.2
Free Hb (g/dL)	0.02 ± 0.01	0.03 ± 0.02	0.04 ± 0.02	0.01 ± 0.004

Data are expressed as the mean ± standard deviation.

The HbV group does not show a significant reduction in the NO_2_^−^ level. In addition, creatinine levels are maintained within normal ranges. There is also no significant increase in free Hb levels.

*Hb* hemoglobin.

### Fetus survival after postpartum hemorrhage

The survival rates of the fetuses after PPH in the left side of the bicornate uterus was 38 ± 42%, 56 ± 49%, and 79 ± 40% in the HES, HbV, and RBC/PPP groups, respectively. There were no significant differences in survival rates among the three groups.

## Discussion

This study developed a severe PPH model after caesarean section using pregnant rabbits. Total haemorrhage was approximately at least 50 mL/kg and led to severe PPH that became lethal within a few hours in the absence of any fluid resuscitation. Using the current PPH model, we demonstrated that HbVs helped to maintain hemodynamics during severe PPH. Consequently, maternal prognosis was better in HbV infusion than in HES infusion alone. Similarly, RBC and PPP transfusion achieved excellent prognosis. The pregnant rabbits receiving RBC transfusion achieved a target value of at least 8.0 g/dL for the Hb concentration, which is recommended after RBC transfusion for PPH^29^. Further, HbV infusion gradually increased the Hb concentration similar to that in transfusion. In contrast to the change in Hb levels, Hct levels were lower in the HbV group than in the RBC/PPP group. The HbV particles used in this study did not affect the Hct level because their particle size (particle diameter: 250–280 nm) was extremely small^30^. To the best of our knowledge, this is the first report of an appropriate animal model requiring massive transfusion according to actual clinical practice.

HbV infusion maintained the CtO_2_ levels to those of the systemic tissues throughout the experiment. Maternal CtO_2_ levels > 8 vol% were consequently achieved, which ensured adequate CtO_2_ for fetal survival^31^. However, HbV infusion yielded a fetus survival rate of only 56%. The in vivo half-life of HbVs (47–72 h) is shorter than that of RBCs^32^, which may affect the maternal prognosis beyond 6 h. In addition, carbonic anhydrase is not incorporated into HbVs; consequently, plasma HCO_3_^-^ levels were significantly lower in the HbV group than in the RBC/PPP group. This might be critical because some studies have reported that low HCO_3_^−^, particularly < 16 mmol/L, independently predicted short-term prognosis^33^. The lactate levels at baseline in the HbV group was statistically higher than that in other groups. The average value of baseline in HbV group was 3.3 mmol/L (< 4.0 mmol/L). This value was clinically within the normal range and did not suggest a lactic acidosis, because the pH levels in the same time point (baseline) were higher than 7.35. In addition, the lactate levels in HbV group were not increased during the experiment. We think that the statistically significant difference in the baseline lactate levels was not clinically relevant difference.

Chemically modified, cell-free HBOCs, including glutaraldehyde-polymerized and PEG-conjugated Hbs, have advanced to clinical trials^34,35^. Nevertheless, such cell-free HBOCs showed toxicities caused by extravasation, oxidative stress, hypertension, and vasoconstriction. In contrast, the current HbVs compartmentalized a concentrated Hb solution in the inner aqueous phase of liposomes, analogous to erythrocytes, leading to reduced toxicities of bared Hb such as hemolysis or acute renal dysfunction^9,18^. HbVs could be stored for at least 12 months and thus may be used in clinical settings where transfusions would be insufficient or unavailable^17^. In the current study, HbV administration had no scavenging effect on plasma NO_2_^-^ levels. HbVs are metabolized by macrophages in the reticuloendothelial system as well as aging RBCs. Cholesterols, phospholipids, and *β*-lipoprotein transiently increase, peaking at 1 or 2 days, and return to the baseline level at 7 days^36^. HbVs do not cross the placental barrier^37^. However, the optimal strategy for improving acute placental hypoxia or shock stress due to hemorrhagic shock is an important issue that needs further research in the management of severe PPH.

Our findings should be interpreted with caution in the context of the principal limitations of this study. First, in this study, the efficacy of HbVs in severe PPH was evaluated in an animal model. As such, we used the transfusion of RBC + PPP (1:1) to maintain hemodynamics in severe PPH, as a positive control. Therefore, the recommended values of fibrinogen (200–400 mg/dL) in PPH was not reached. In addition, albumin preparation alone was not used for a negative control. Thus, the applicability of the findings in humans and the improved protocol for severe PPH need to be further verified. Second, we need to investigate the late toxicities of HbVs beyond the acute setting, such as the maternal effect in the late phase due to huge-volume infusions of HbVs for severe PPH in humans. Some studies, including this study, have reported the safety of huge-volume infusions of HbVs in animal models^36,38^, but not in humans. Third, we did not precisely investigate the effects of HbVs, including intrauterine fetal resuscitation, in this study. A previous study reported the efficacy of HbVs for chronic placental hypoxia and improvement in fetal growth restriction in a rat pre-eclampsia model^37^. However, there are no reports on the efficacy of intrauterine resuscitation for low uteroplacental blood flow due to acute massive peripartum haemorrhage, including placental abruption. In this study, HbV and HES groups showed coagulopathy caused by low levels of PLT and coagulation factors, or the possible effect of HES infusion. In addition, ACT, which relied in the presence of PLT, was significantly prolonged with decreasing PLT counts. Controlling coagulopathy should be further investigated using other blood substitutes with haemostatic capacity, including PLT substitutes^39^, or the combined resuscitation fluids of crystalloids, colloids, or albumin. Lastly, this study had methodological limitations. The measurement of systemic NO_2_^−^ levels did not consistently reflect NO metabolism at the cellular levels.

Despite these limitations, this study provides important baseline evidence in developing initial fluid treatment with HbV infusion for severe PPH. The remarkable resuscitative effect of HbV treatment in the first few hours reduced the elevation of plasma lactate levels. Thus, prompt HbV infusion might help stabilize the patients’ hemodynamics and enable their transportation to secondary or tertiary facilities.

In conclusion, HbVs effectively prevented lethal haemorrhagic shock due to severe PPH in pregnant rabbit models. These findings support that these blood substitutes are possible alternative modalities for the initial and immediate treatment of severe PPH, particularly in institutions with limited availability of blood products, until transportation to a secondary or tertiary facility.

## Supplementary Information

Supplementary Information.

## References

[1] Say L (2014). Global causes of maternal death: A WHO systematic analysis. Lancet Glob. Health..

[2] O’Brien KL, Shainker SA, Lockhart EL (2018). Transfusion management of obstetric hemorrhage. Transfus. Med. Rev..

[3] Weeks A (2015). The prevention and treatment of postpartum haemorrhage: What do we know, and where do we go to next?. BJOG.

[4] Committee on Practice Bulletins Obstetrics (2017). Practice Bulletin No. 183: Postpartum hemorrhage. Obstet. Gynecol..

[5] Wang Y (2020). Impact of COVID-19 on blood centres in Zhejiang Province China. Vox. Sang..

[6] Baron DM (2020). Patient blood management during the COVID-19 pandemic: A narrative review. Anaesthesia.

[7] Sakai H (2017). Overview of potential clinical applications of hemoglobin vesicles (HbV) as artificial red cells, evidenced by preclinical studies of the Academic Research Consortium. J. Funct. Biomater..

[8] Natanson C, Kern SJ, Lurie P, Banks SM, Wolfe SM (2008). Cell-free hemoglobin-based blood substitutes and risk of myocardial infarction and death: A meta-analysis. JAMA.

[9] Sakai H, Sou K, Tsuchida E (2009). Hemoglobin-vesicles as an artificial oxygen carrier. Methods Enzymol..

[10] Taguchi K (2010). Hepatically-metabolized and -excreted artificial oxygen carrier, hemoglobin vesicles, can be safely used under conditions of hepatic impairment. Toxicol. Appl. Pharmacol..

[11] Takahashi D (2011). Phagocytosis of liposome particles by rat splenic immature monocytes makes them transiently and highly immunosuppressive in ex vivo culture conditions. J. Pharmacol. Exp. Ther..

[12] Sakai H, Suzuki Y, Sou K, Kano M (2012). Cardiopulmonary hemodynamic responses to the small injection of hemoglobin vesicles (artificial oxygen carriers) in miniature pigs. J. Biomed. Mater. Res. A..

[13] Taguchi K (2009). Hemoglobin vesicles, polyethylene glycol (PEG)ylated liposomes developed as a red blood cell substitute, do not induce the accelerated blood clearance phenomenon in mice. Drug. Metab. Dispos..

[14] Sakai H (2004). Physiological capacity of the reticuloendothelial system for the degradation of hemoglobin vesicles (artificial oxygen carriers) after massive intravenous doses by daily repeated infusions for 14 days. J. Pharmacol. Exp. Ther..

[15] Seishi Y, Horinouchi H, Sakai H, Kobayashi K (2012). Effect of the cellular-type artificial oxygen carrier hemoglobin vesicle as a resuscitative fluid for prehospital treatment: Experiments in a rat uncontrolled hemorrhagic shock model. Shock.

[16] Hagisawa K (2018). Efficacy of resuscitative transfusion with hemoglobin vesicles in the treatment of massive hemorrhage in rabbits with thrombocytopenic coagulopathy and its effect on hemostasis by platelet transfusion. Shock.

[17] Tokuno M, Taguchi K, Yamasaki K, Sakai H, Otagiri M (2016). Long-term stored hemoglobin-vesicles, a cellular type of hemoglobin-based oxygen carrier, has resuscitative effects comparable to that for fresh red blood cells in a rat model with massive hemorrhage without post-transfusion lung injury. PLoS ONE.

[18] Sakai H, Sou K, Horinouchi H, Kobayashi K, Tsuchida E (2008). Haemoglobin-vesicles as artificial oxygen carriers: Present situation and future visions. J. Intern. Med..

[19] Waters JH (2014). Role of the massive transfusion protocol in the management of haemorrhagic shock. Br. J. Anaesth..

[20] Yu YH, Gong SP, Sheng C, Zhao KS, Lodato RF, Wang CH (2009). Increased survival with hypotensive resuscitation in a rabbit model of uncontrolled hemorrhagic shock in pregnancy. Resuscitation.

[21] Kilkenny C, Browne WJ, Cuthill IC, Emerson M, Altman DG (2010). Improving bioscience research reporting: The ARRIVE Guidelines for Reporting Animal Research. PLoS Biol..

[22] du Sert NP (2020). The ARRIVE guidelines 2.0: Updated guidelines for reporting animal research. PLoS Biol..

[23] Sakai H, Yuasa M, Takeoka S, Tsuchida E (2000). Synthesis and physicochemical characterization of a series of hemoglobin-based oxygen carriers: Objective comparison between cellular and acellular types. Bioconjugate.

[24] Kawaguchi R (2019). Guidelines for office gynecology in Japan: Japan Society of Obstetrics and Gynecology (JSOG) and Japan Association of Obstetricians and Gynecologists (JAOG) 2017 edition. J. Obstet. Gynaecol. Res..

[25] Diehl KH (2001). A good practice guide to the administration of substances and removal of blood, including routes and volumes. J. Appl. Toxicol..

[26] Cannon JW (2018). Hemorrhagic shock. N. Engl. J. Med..

[27] Finter S (2010). Resuscitation fluid use in critically ill adults: An international cross-sectional study in 391 intensive care units. Crit. Care..

[28] Faul F, Erdfelder E, Lang AG, Buchner AG (2007). G*Power 3: A flexible statistical power analysis program for the social, behavioral, and biomedical sciences. Behav. Res. Methods..

[29] Jansen AJ, van Rhenen DJ, Steegers EA, Duvekot JJ (2005). Postpartum hemorrhage and transfusion of blood and blood components. Obstet. Gynecol. Surv..

[30] Barroso J (2018). Safety evaluation of a lyophilized platelet-derived hemostatic product. Transfusion.

[31] Moon PF, Bliss SP, Posner LP, Erb HN, Nathanielsz PW (2001). Fetal oxygen content is restored after maternal hemorrhage and fluid replacement with polymerized bovine hemoglobin, but not with hetastarch, in pregnant sheep. Anesth. Analg..

[32] Taguchi K, Watanabe H (2012). A fourteen-day observation and pharmacokinetic evaluation after a massive intravenous infusion of hemoglobin-vesicles (artificial oxygen carriers) in cynomolgus monkeys. J. Drug. Metab. Toxicol..

[33] Wigger O (2018). Baseline serum bicarbonate levels independently predict short-term mortality in critically ill patients with ischaemic cardiogenic shock. Eur. Heart J. Acute Cardiovasc. Care..

[34] Olofsson C (2006). A multicenter clinical study of the safety and activity of maleimide-polyethylene glycol-modified hemoglobin (Hemospan) in patients undergoing major orthopedic surgery. Anesthesiology.

[35] Jahr JS, Mackenzie C, Pearce LB, Pitman A, Greenburg AG (2008). HBOC-201 as an alternative to blood transfusion: Efficacy and safety evaluation in a multicenter phase III trial in elective orthopedic surgery. J. Trauma..

[36] Yamamoto M (2012). Fluid resuscitation of hemorrhagic shock with hemoglobin vesicles in Beagle dogs: pilot study. Artif. Cells Blood Substit. Immobil. Biotechnol..

[37] Li H (2015). Artificial oxygen carriers rescue placental hypoxia and improve fetal development in the rat pre-eclampsia model. Sci. Rep..

[38] Sakai H (2009). Fluid resuscitation with artificial oxygen carriers in hemorrhaged rats: Profiles of hemoglobin-vesicle degradation and hematopoiesis for 14 days. Shock.

[39] Hagisawa K (2019). Combination therapy using fibrinogen γ-chain peptide-coated, ADP-encapsulated liposomes and hemoglobin vesicles for trauma-induced massive hemorrhage in thrombocytopenic rabbits. Transfusion.

