# Supplementary material for: Resuscitative efficacy of hemoglobin vesicles for severe postpartum hemorrhage in pregnant rabbits
Source: Sci Rep. 2021 Nov 16;11:22367. doi: 10.1038/s41598-021-01835-w (PMC8595665; doi:10.1038/s41598-021-01835-w)
Supplement: Supplementary file 1 — Supplementary Information. [file 41598_2021_1835_MOESM1_ESM.pdf]

Supplemental Information

Resuscitative efficacy of hemoglobin vesicles for severe postpartum hemorrhage in pregnant rabbits

Hiroki Ishibashi, Kohsuke Hagisawa, Manabu Kinoshita, Yukako Yuki, Morikazu Miyamoto, Tomoko Kure, Hiromi Sakai, Daizoh Saito, Katsuo Terui, Masashi Takano

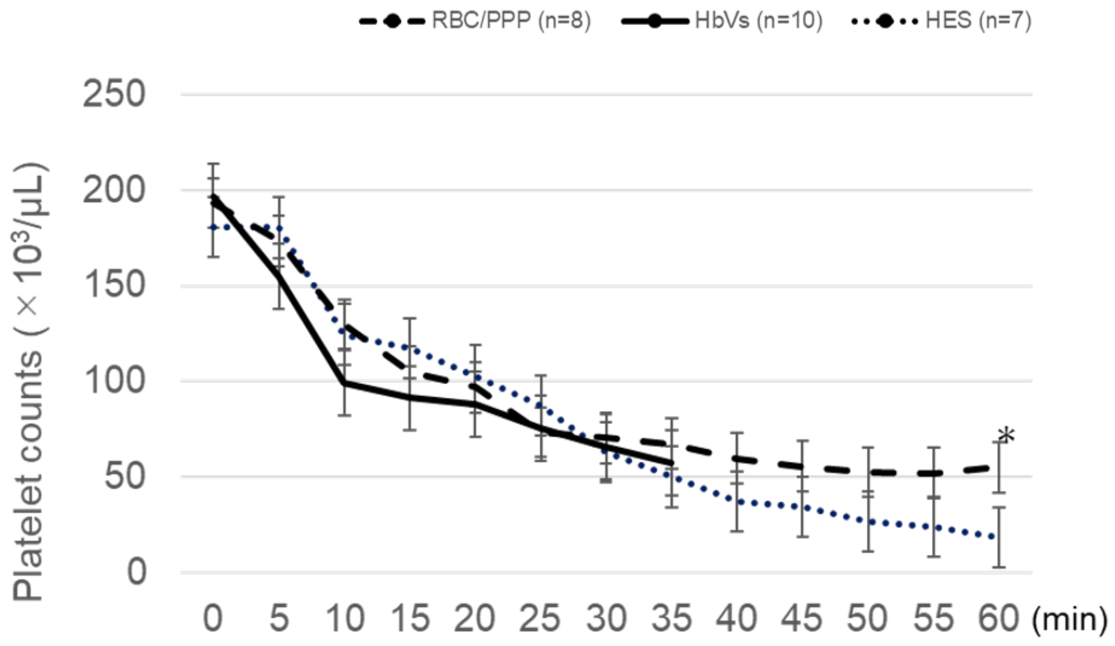

Supplementary Figure S1. Changes in platelet counts.

Although the platelet count was lower in the RBC/PPP group, it was significantly higher than that in the HES group at the end of the experiment. * $P < 0.05$, value significantly different from the other group. Data shown are the mean ± SD.

Supplementary Note. The items based on The ARRIVE essential 10.

1. Study design

Study design was described in the paragraphs of “**Surgical procedures,” “Severe postpartum hemorrhage model,”** and **“Fluid resuscitation with hemoglobin vesicles, RBCs, or 6% HES”** in **the Methods**.

2. Sample size

The sample size was calculated as follows: the effect size was defined as 0.6, and we permitted a type 1 error of α=0.05 and type 2 error of β=0.2. The results of the sample size analysis showed a need for 30 rats (10 rats per group). However, the number of positive and control rats was reduced to the minimum for animal protection. Finally, we decided the sample size of each group, as described in the manuscript.

3. Inclusion and exclusion criteria

Inclusion and exclusion criteria were not set in this study. The results for each analysis are described in the **Results** section.

4. Randomization

Rabbits were randomly classified into three groups based on the resuscitation fluid used. Randomization was performed using the random function in Microsoft Excel.

5. Blinding

In this study, the investigator could not be blinded due to the difference in treatment.

6. Outcome measures

These parameters were described in the **Methods** and **Results**.

7. Statistical methods

Statistical methods were described in under the heading "**Statistical analysis**" in the **Methods**.

8. Experimental animals.

Experimental animals were described under the heading "**Animal management**" and "**Preparation of allogenic RBCs**" in the **Methods**.

9. Experimental procedures

Experimental procedures were described under the heading **"Surgical procedures," "Severe postpartum hemorrhage model,"** and **"Fluid resuscitation with hemoglobin vesicles, RBCs, or 6% HES"** in **the Methods (Figure 6)**.

10. Results

The investigated parameters in this experiment were shown in the **Results**.
